# Confining the Concept of Vascular Depression to Late-Onset Depression: A Meta-Analysis of MRI-Defined Hyperintensity Burden in Major Depressive Disorder and Bipolar Disorder

**DOI:** 10.3389/fpsyg.2019.01241

**Published:** 2019-05-31

**Authors:** Katharina I. Salo, Jana Scharfen, Isabelle D. Wilden, Ricarda I. Schubotz, Heinz Holling

**Affiliations:** Department of Psychology and Sports Sciences, Institute of Psychology, Westfälische Wilhelms-Universität, Münster, Germany

**Keywords:** white matter hyperintensities (WMH), leukoaraiosis, major depressive disorder, bipolar disorder, depression, cerebrovascular pathology, hyperintense lesions

## Abstract

**Background:** The *vascular depression hypothesis* emphasizes the significance of vascular lesions in late-life depression. At present, no meta-analytic model has investigated whether a difference in hyperintensity burden compared to controls between late-life and late-onset depression is evident. By including a substantial number of studies, focusing on a meaningful outcome measure, and considering several moderating and control variables, the present meta-analysis investigates the severity of hyperintensity burden in major depressive disorder (MDD) and bipolar disorder (BD). A major focus of the present meta-analysis refers to the role of age at illness onset. It is analyzed whether late-onset rather than late-life depression characterizes vascular depression.

**Method:** In total, 68 studies were included in the meta-analysis and a multilevel random effects model was calculated using Hedges' *g* as the effect size measure.

**Results:** The severity of hyperintensity burden was significantly greater in the patient group compared to the control group. This effect was evident regarding the whole patient group (*g* = 0.229) as well as both depression subgroups, with a significantly greater effect in BD (*g* = 0.374) compared to MDD (*g* = 0.189). Hyperintensity burden was more pronounced in late-onset depression than in early-onset depression or late-life depression. A considerable heterogeneity between the included studies was observed, which is reflected by the large variability in effects sizes.

**Conclusion:** In conclusion, the present meta-analysis underscores the association of hyperintensities with MDD and BD. Especially late-onset depression is associated with an increased hyperintensity burden, which is in line with the *vascular depression hypothesis*. The results suggest that it might be more feasible to confine the concept of vascular depression specifically to late-onset depression as opposed to late-life depression. Further research is needed to understand the causal mechanisms that might underlie the relation between hyperintensity burden and depression.

## Introduction

### Rationale

Major depressive disorder (MDD) and bipolar disorder (BD) have shown to be associated with hyperintense lesions in various MRI studies. Research in this area focuses predominantly on white matter hyperintensities, especially in later life (van Agtmaal et al., 2017). However, these lesions occur in both gray and white matter (Greenwald et al., 1996, 2001; Steffens et al., 2001; Beyer et al., 2009). There is a multitude of studies examining the relationship between hyperintensities and unipolar and bipolar depression, but findings reveal considerable inconsistencies with regard to whether this association exists at all (Lee et al., 2003; Sassi et al., 2003; Dalby et al., 2010), and whether it depends on lesion location (Krishnan et al., 2006). Furthermore, meta-analytic reviews do not consistently coincide and in several cases relevant moderators, e.g., lesion location or age, are not taken into account. To date, no meta-analytic model has investigated whether there is a difference in hyperintensity burden compared to controls between late-life and late-onset depression. In terms of the outcome measure, the majority of prior meta-analyses have focused on prevalence rates rather than severity of hyperintensities. In the case of BD, there is no meta-analysis that has investigated the difference in the severity of hyperintensity burden in comparison to healthy controls. Thus, the goal of the present work was to provide a meta-analytic update on the association of hyperintensities with MDD and BD by including a comprehensive number of studies, elucidating the role of age at illness onset and lesion location, and defining the severity of hyperintensities as an outcome measure.

#### Hyperintense Lesions

Hyperintensities are assumed to reflect silent lesions of vascular origin in the brain (Farkas et al., 2006). Their relaxation properties in magnetic resonance imaging (MRI) make hyperintensities appearing as particularly bright signals, i.e., areas of increased signal intensity, on T2-weighted MRI images (Pantoni and Garcia, 1997). These lesions are not restricted to pathologic conditions affecting the central nervous system such as Alzheimer's disease, Parkinson's disease, or small vessel disease (Radanovic et al., 2013; Compta et al., 2016; Foo et al., 2016; Li et al., 2016). It is a phenomenon that is frequently observed in aging (Hendrie et al., 1989; Breteler et al., 1994; Gattringer et al., 2012). The prevalence rates reported by several studies vary, which may be due to different MRI procedures or rating scales (Mäntylä et al., 1997), but altogether hyperintensities can be considered as a rather common phenomenon in the elderly. For example, a population-based study by de Leeuw et al. (2012) reported that only 5% of the study sample aged between 60 and 90 were free of any hyperintense white matter lesions. However, the clinical relevance of hyperintense lesions should not be underestimated. Particularly, white matter hyperintensities (WMH) are frequently associated with cognitive deficits in different domains. As demonstrated by the meta-analysis by Kloppenborg et al. (2014) the presence of white matter hyperintensities is related to cognitive deficits regarding general intelligence, memory, processing speed, attention, and executive functions. Moreover, they found that WMH progression was associated with greater cognitive decline over time in executive functions, attention, and general intelligence.

The origin of hyperintense lesions may vary across conditions, and the exact causes remain to be conclusively determined. However, the prevalence of hyperintensities increases in later life, which can be explained by the fact that old age is more commonly associated with various changes in the vascular system both on structural and functional levels (Marín, 1995; Kovacic et al., 2011), and considering that vascular or ischemic pathology is assumed to account for the majority of hyperintensities (Pantoni and Garcia, 1997; Bakker et al., 1999; Farkas et al., 2006). Furthermore, diseases with vascular impact such as hypertension and diabetes mellitus increase with age (Kearney et al., 2005; Shaw et al., 2010) and are associated with hyperintensities (Longstreth et al., 1996; Habes et al., 2016). Post-mortem studies reveal a variety of etiologic patterns for hyperintensities. Findings most often reported as being associated with hyperintense lesions include myelin rarefaction, arteriosclerosis, dilated perivascular spaces, vascular ectasia, ependymal loss, and cerebral ischemia (Awad et al., 1986; Kirkpatrick and Hayman, 1987; Fazekas et al., 1991; Scheltens et al., 1995). Similar relations were found in studies investigating the neuropathological substrates of hyperintensities in depressed subjects (Thomas et al., 2002, 2003). While there is evidence for WMH as preceding depression or depressive symptoms (Teodorczuk et al., 2007, 2012; Firbank et al., 2012; Qiu et al., 2017), other studies did not find such a pattern (Versluis et al., 2006; Dotson et al., 2013). Therefore, a potential causal relation between hyperintensities and depression remains unclear.

#### The Vascular Depression Hypothesis

Diseases involving cerebrovascular symptomatology are assumed to function as a predisposing, triggering or perpetuating factor for some depressive syndromes in the elderly (Alexopoulos et al., 1997). The *vascular depression hypothesis* is based on the association between depression and vascular pathology or vascular risk factors and their behavioral correlates. Vascular compared to non-vascular depression can be assumed to be associated, among others, with old age and old age at illness onset (Krishnan et al., 1997). More recently, Krishnan et al. (2004) introduced the term *subcortical ischemic depression* (SID) to describe vascular-related depression. The authors identified SID on the basis of deep white matter hyperintensity (DWMH) and subcortical gray matter hyperintensity (SCGMH) ratings. They found that age, lassitude, and a history of hypertension were associated with SID. A study investigating the internal validity of the vascular depression concept identified DWMH burden as the most specific and sensitive factor for distinguishing vascular from non-vascular late-life depression (Sneed et al., 2008). Further evidence regarding the external validity of vascular depression is established by studies which found that the vascular subtype is associated with a more severe psychomotor retardation (Pimontel et al., 2013) and lower response rates to antidepressant medication (Sneed et al., 2011) than the non-vascular subtype.

### Objectives

The present meta-analysis aims to increase insight into the association of hyperintensities and depression mainly in view of two key aspects. Firstly, relevant moderators were examined, which is especially crucial with regard to age at illness onset, lesion location and investigating MDD as well as BD. The second key aspect drew attention to the severity (as opposed to the dichotomously defined presence vs. non-presence, i.e., frequency) of hyperintensities. The rationale for why these issues are emphasized is described below. In addition, several methodological aspects were considered during different research stages (i.e., exclusion criteria, potential confounders, publication bias), which have not consistently been applied by previous meta-analytic studies regarding this topic.

Notably, though age at illness onset has been shown to moderate the association between hyperintensities and depression disorders in several studies (Lesser et al., 1996; Lloyd et al., 2004; Tamashiro et al., 2008; Delaloye et al., 2010), in current meta-analytic research on late-life depression its moderating role is not consistently adhered to. A categorical distinction can be made between early-onset and late-onset depression according to the age at illness onset. Late-life depression as such which geriatric depression is commonly referred to can comprise both an early and a late illness onset. Cut-offs to define late-life depression or to differentiate between an early and a late illness onset usually vary from 50 to 65 years (Aizenstein et al., 2016). Alexopoulos et al. (1997) proposed the *vascular depression hypothesis* which is related to late-life depressive syndromes. While it focuses on late-life depression, it explicitly includes early-onset depression in later life (Taylor et al., 2013a). To investigate if late-onset rather than late-life depression might be more effective to determine vascular depression, the present meta-analysis defined late-onset depression as a distinct category in addition to late-life depression. This was done to account for the crucial role of age at illness onset, which the commonly used categorization of late-life depression does not take into consideration. Moreover, two depression types, unipolar and bipolar, were investigated. In the case of BD, the most recent meta-analysis is from 2009 (Beyer et al., 2009), which makes a meta-analytical update crucial. A further emphasis was placed on lesion location in terms of a possible moderator, as previous meta-analytic results show inconsistencies with regard to the association of hyperintensities with MDD or BD in different lesion locations.

The second major characteristic of the present meta-analysis is its focus on the severity of hyperintensities instead of the frequency of hyperintensities (i.e., dichotomous categorization as present or not). This is particularly relevant, since several studies found that an increase in the severity of white matter hyperintensities (WMH) is associated with more pronounced cognitive impairment (de Groot et al., 2001; Murata et al., 2001; Chen Y. F. et al., 2006). Therefore, the severity of hyperintensity burden seems to be of clinical significance with respect to depressive symptomatology. In addition, hyperintensities are frequently observed in aging irrespective of pathological conditions (Hendrie et al., 1989; Breteler et al., 1994; Gattringer et al., 2012). In this regard, comparing the severity rather than the frequency of hyperintensities between depressed patients and healthy controls seems more comprehensive.

With respect to methodological issues, the present meta-analysis aims at extending previous findings by including a substantial number of studies, applying an appropriate statistical model—namely a multilevel random effects model to account for dependencies between outcomes and evaluating heterogeneity of the effect—and by conducting statistical analyses to control for several demographic and methodological differences between studies. Furthermore, in order to account for clinical validity, only studies that provide explicit diagnoses of MDD or BD according to DSM or ICD criteria are included.

### Review of the Meta-Analytic State of Research

To date, nine meta-analyses have investigated the association of hyperintensities with MDD and BD (Altshuler et al., 1995; Videbech, 1997; Herrmann et al., 2008; Kempton et al., 2008, 2011; Beyer et al., 2009; Anorne et al., 2012; Wang et al., 2014; van Agtmaal et al., 2017). Of these meta-analyses, five studies investigated the association with MDD (Herrmann et al., 2008; Kempton et al., 2011; Anorne et al., 2012; Wang et al., 2014; van Agtmaal et al., 2017), three with BD (Altshuler et al., 1995; Kempton et al., 2008; Beyer et al., 2009), and one with MDD and BD separately (Videbech, 1997). These prior studies provide important results on the association between hyperintensities and depression and are a good starting point for further meta-analytic research.

Each meta-analysis renders some evidence in favor of increased hyperintensities in unipolar and bipolar depression. Investigating lesion location, Kempton et al. (2008) and Beyer et al. (2009) found a significantly higher prevalence of hyperintensities in BD subjects compared to control subjects regarding deep white matter hyperintensities (DWMH) but not periventricular hyperintensities (PVH). For subcortical gray matter hyperintensities (SCGMH), only the meta-analysis by Beyer et al. (2009) found an increased prevalence in BD. Altshuler et al. (1995) did not differentiate between lesion locations, but found a higher frequency of hyperintensities in BD subjects compared to controls. Wang et al. (2014) report that DWMH, but not PVH or overall WMH, were associated with MDD. Kempton et al. (2011), on the contrary, found a significant association of MDD with PVH but not with DWMH. Focusing on late-life MDD, Herrmann et al. (2008) report significantly increased PVH and DWMH. van Agtmaal et al. (2017) demonstrated that overall WMH were more frequent in late-life depression than in controls. The meta-analysis by Anorne et al. (2012) reports an increased volume of WMH in MDD patients compared to controls. Videbech (1997) reports a higher risk for hyperintensities in both MDD and BD not specifying lesion location. In summary, in the case of BD, meta-analyses agree on a higher prevalence of DWMH compared to controls while no effect was observed regarding PVH. For SCGMH, meta-analyses do not coincide. In the case of MDD, results are diverging with respect to DWMH and PVH. The prevalence of SCGMH in MDD was only investigated by Kempton et al. (2011) who report a higher frequency of MDD patients with SCGMH than controls.

Importantly, with regard to examining the difference in the severity of hyperintensity burden between BD patients and controls (instead of the frequency), there is no meta-analytic research yet. For MDD, the severity of hyperintensity burden was investigated in three prior meta-analyses (Herrmann et al., 2008; Kempton et al., 2011; Anorne et al., 2012) but the number of studies included in the analyses is limited. The meta-analysis by Anorne et al. (2012) comprises four studies and the meta-analysis by Kempton et al. (2011) nine. In the meta-analysis by Herrmann et al. (2008) 13 studies on late-life depression and five on late-onset depression were included. Thus, the severity of hyperintensity burden in BD patients compared to controls needs to be scrutinized in meta-analytic research. In the case of MDD, the hyperintensity severity in different onset subgroups needs further evaluation, as the meta-analysis by Herrmann et al. (2008) is the only study that investigated late-onset depression as opposed to late-life depression. Taking the year of publication into consideration as well as the total number of included studies, a meta-analytic update is essential.

With respect to methodological issues, the number of included studies generally ranges from four (Anorne et al., 2012) to 38 studies in the meta-analysis by van Agtmaal et al. (2017). Here, however, in the analysis of studies where an explicit MDD diagnosis was provided, only 16 studies were included (as opposed to defining depression as the mere presence of depressive symptoms assessed by questionnaires). Strikingly, a depression diagnosis was not necessarily part of the inclusion criteria of previous meta-analyses. For example, the meta-analysis conducted by Wang et al. (2014) investigated hyperintensities in patients with MDD diagnosis and subjects exhibiting depressive symptoms without distinguishing between these two conditions. Another aspect regarding inclusion criteria concerns the selectivity of the patient sample according to comorbidities. The meta-analysis by van Agtmaal et al. (2017), for instance, comprises patient samples selected according to specific disease factors (e.g., post-stroke depression). In such highly selected samples it cannot be excluded that the association between depression and hyperintensities is confounded with the effect of this underlying factor. Concerning statistical analysis, a random effects model seems reasonable and, in addition, a multilevel analysis would be appropriate to account for dependencies between outcomes within the same study or sample. This heterogeneity, however, was not accounted for in the meta-analyses by Altshuler et al. (1995), Beyer et al. (2009), and Videbech (1997), and none of the meta-analyses applied a multilevel model.

Taken together, the different meta-analyses elucidate important aspects of the association between hyperintensities and depression, for example, with regard to the role of different lesion locations. Further strengths of these meta-analyses refer, among others, to the careful handling of different outcome measures in the primary studies. That is, accounting for differences in lesion assessment, for example, by selectively excluding inappropriate outcomes or pooling primary studies according to methodological aspects (e.g., van Agtmaal et al., 2017). Therefore, these meta-analyses represent a meaningful starting point for a meta-analytic update on the association of hyperintensities with MDD and BD. Further meta-analytic investigation seems reasonable since prior meta-analyses differ in overall effect sizes and partly report contradicting results with respect to lesion location. Moreover, taking into account the heterogeneity between primary studies, several demographic and methodological variables should be controlled for to exclude confounding. Concerning moderating factors, investigating the role of late-onset depression as opposed to late-life depression might increase insight into the feasibility of the current conceptualization of vascular depression. In addition, the above delineated methodological issues might potentially limit the validity of previous findings, which can be resolved by a meta-analytic update.

### Hypotheses

The severity of hyperintensity burden in MDD and BD compared to healthy controls has been explored. Possible confounders and moderators of this association have been derived from the research outlined above and from methodological considerations. Age at illness onset defined by group (early-onset vs. late-onset) has been assumed to act as a moderating variable not only in MDD (Herrmann et al., 2008), but also in BD (Tamashiro et al., 2008). Further, the role of lesion location has been examined. There is evidence for hyperintensities to occur in the deep white matter (DWMH), in periventricular areas (PVH) and in subcortical gray matter structures (SCGMH) in both MDD and BD (Greenwald et al., 1996; McDonald et al., 1999; Beyer et al., 2009). Thus, the hypotheses drawn from considerations on the current state of research were as follows:

1. Hyperintensity burden is higher in subjects with depression (MDD and BD) than in healthy control subjects. This association is evident in both MDD and BD and in different lesion locations (DWMH, PVH, SCGMH) in both disorders.
2. Late-onset depression is associated with a higher hyperintensity burden than early-onset depression in both MDD and BD.

## Method

### Search Strategy

**Figure 1:**
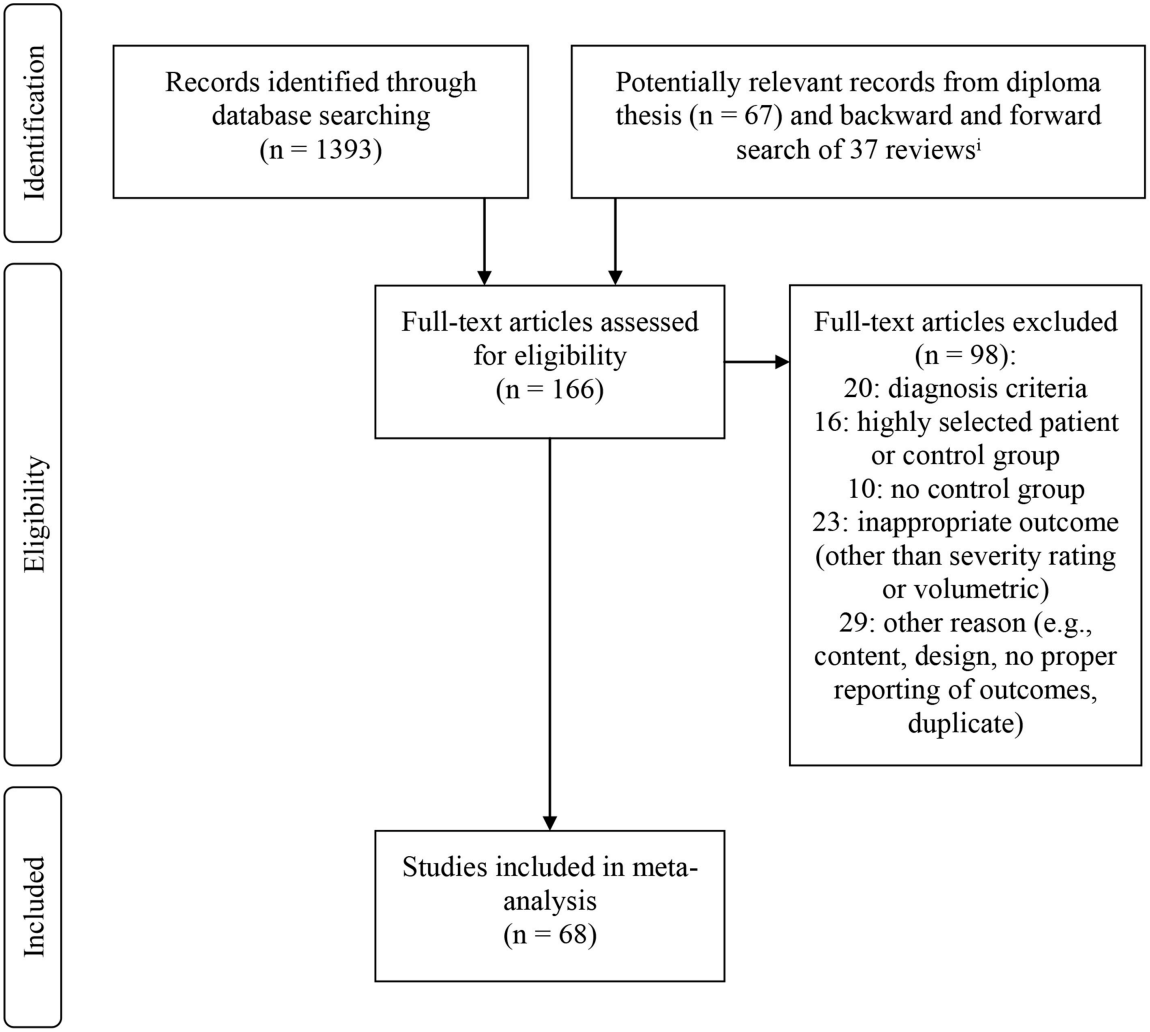
Flow chart displaying inclusion and exclusion of studies in meta-analysis. ^*i*^All identified titles were screened for relevance. If titles were regarded as relevant, abstracts were screened. If studies were regarded as potentially eligible on the basis of the abstract, full-text articles were retrieved.

The starting point for the present meta-analysis was an unpublished diploma thesis by Isabelle D. Wilden (Mokwa, 2013). To also include newer publications, a database literature search for studies published since January 2012 was conducted using the search term: (Leukoaraiosis OR Hyperintens^*^ OR Lesion^*^ OR Leukoencephalopath^*^) AND (Hypoman^*^ OR Depress^*^ OR Unipolar^*^ OR Bipolar^*^ OR Mania OR Manic OR Affectiv^*^) AND (mri OR “Magnetic Resonance Imaging”). The databases which were included in this search were: MEDLINE (PubMed), PsycINFO, Academic Search Premier, PsycARTICLES, PsycCritiques, PsycBOOKS, PSYNDEX, and ProQuest. A total of 1,393 titles were screened, and if regarded as relevant, abstracts were reviewed. Of all potentially relevant studies, full texts were examined. In addition, backward and forward search was conducted for 37 reviews including the prior meta-analyses. Database search was terminated in March 2017. Backward and forward search was completed in July 2017. Figure 1 displays the result of the search procedure. In total, 68 studies were included in the meta-analysis.

### Inclusion and Exclusion Criteria

The predefined inclusion criteria were as follows: Firstly, diagnosis of either MDD or BD must be established in the patient sample. Diagnosis must be made according to either *Diagnostic and Statistical Manual of Mental Disorders* (DSM) or *International Classification of Diseases* (World Health Organization, 1992), excluding classification systems older than DSM-III-R (American Psychiatric Association, 1987) to ensure clinical validity. The diagnosis criterion therefore excludes studies in which diagnosis is either based on self-report of prior MDD or BD diagnosis, based on self-report of having been in psychotherapeutic treatment due to depressive or manic symptomatology, based on the prescription of antidepressants or mood stabilizing medication, or based on cut-off scores of inventories assessing depressive or bipolar symptomatology. Secondly, the severity of hyperintensity burden must be assessed in the patient sample and in a healthy control group. A life-time history of affective disorders must be excluded in control subjects to avoid confounding. Thirdly, hyperintensity burden must be assessed via MRI in both groups. Lastly, the studies must be published in English.

Furthermore, studies were excluded if, firstly, the patient or control group was selected according to a specific disease factor (e.g., post-stroke depression and migraine) or otherwise highly selected (e.g., craniocerebral injury), and, secondly, if neurodegenerative or neuroinflammatory diseases (e.g., Alzheimer's disease and multiple sclerosis) were included in either the patient or control group since hyperintensities are common among these pathological conditions as well.

### Coding Scheme

The coding scheme was developed on the basis of the current state of research (Data Sheet 1) and methodological considerations on possible confounders. The variables were assigned to four categories. The first category comprised variables that refer to the main effects. These were the variables disorder, hyperintensity burden, lesion location and illness onset defined by group (i.e., early-onset, late-onset, late-life, and not specified). The second category included several demographic control variables. These were age, sex (%), smoking (%), antidepressant medication (%), severity of depression, age at illness onset, hypertension (%), and diabetes mellitus (%). Methodological control variables were assigned to the third category which comprised the variables matching, blinding, year of publication, hyperintensities as primary vs. secondary research interest, assessment of hyperintensities (i.e., visual rating scale, automated, semi-automated), type of rating scale used, and outcome measure (i.e., severity rating vs. lesion volume). In short, automated lesion assessment describes the fully automated assessment of lesion volume via different adapted algorithms applied to the MRI scans. An automated segmentation process is operated using individually designed or at hand software. Predefined segmentation criteria are incorporated into the algorithms to identify lesion volume by differentiating between different kinds of brain tissue. In semi-automated processes, automatically pre-labeled MRI scans are additionally inspected by a researcher who manually outlines relevant lesions. Either lesion volume or severity ratings according to visual rating scales can be inferred. The fourth category of control variables incorporated differences in MRI procedures, namely the variables tesla, plane, slice thickness, and interslice gap.

Since the outcome measures of hyperintensity burden (e.g., volumetric vs. rating scale) were not homogenous between studies, the influence of the demographic variables on lesion load in the patient and the control sample could not directly be calculated. Furthermore, to investigate possible confounding it is necessary to determine if a difference in these variables between the patient and the control group moderates the association between depression and hyperintensity burden. For example, with increasing age the prevalence of hyperintensities increases. Therefore, it has to be excluded that differences in age between the patient and the control group might influence the difference in hyperintensity burden between patients and controls. Thus, to investigate the impact of these variables on the effect size measure (i.e., difference in hyperintensity burden), the differences between the patient and the control group were calculated. Concretely, the variables age, sex, smoking, hypertension, and diabetes were transformed into a new variable defined as the difference between the patient and the control group. For age, the years were subtracted. In the case of prevalence data (sex, smoking, hypertension, and diabetes), the percentages were subtracted. The influence of the difference in these variables between patients and controls on the standardized mean difference (hyperintensity burden) was analyzed. Taken together, investigating the influence of the differences in these demographic variables on the differences in lesion load (i.e., the effect size measure) allows controlling for possible confounding irrespective of different outcome measures between the studies.

In cases where samples overlapped between studies these studies were coded as one study. In this way, it was controlled for dependencies between outcomes due to dependencies between samples. Consequently, the studies by Potter et al. (2007), Taylor et al. (2007), and Taylor et al. (2008) were coded as different samples from the same study. In addition, the samples from the studies by McDonald et al. (1991) and McDonald et al. (1999) were coded as belonging to the same study.

Concerning lesion location, the different subcortical structures or white matter lesion locations were assigned to the categories OWMH, DWMH, PVH, and SCGMH.

All studies included in the meta-analysis were coded according to the above delineated coding scheme. If data on moderator or control variables was not directly reported, it was inferred from the given information where possible. Either lesion volume or severity ratings assessed via visual rating scales (Fazekas et al., 1987; Coffey et al., 1990; Scheltens et al., 1993) were regarded as indicators for the severity of hyperintensity burden. When outcome data for hyperintensities was not provided in an applicable form, authors of eligible studies were contacted. One of six authors replied and the study was included in the analyses.

As the meta-analysis represents an investigation of cross-sectional data and no intervention was examined (which excludes selection bias, performance bias, and attrition bias), blinding of the researcher in cases of visual severity ratings or semi-automated lesion assessment procedures was regarded as the primary control variable for assessing potential risk of bias (i.e., detection bias). Reporting bias is considered minimal, as highly selected samples were not included in the analyses (see inclusion and exclusion criteria). In addition, the risk of publication bias has been examined in multiple ways (see statistical analyses).

### Effect Size Measure

Hedges' *g* was chosen as the effect size measure since it represents an appropriate calculation to determine the standardized mean difference between two groups. Hedges' *g* has the advantage of applying a correction term *J* to Cohen's *d* so that it is corrected for possible overestimation of the effect size due to small sample size. In the present meta-analysis, Hedges' *g* displays the difference in hyperintensity burden between the patient and the control group. Hedges' *g* is defined by the following formula (Hedges, 1981):

$$\begin{array}{l} g=\frac{{\bar{x}}_{1}-{\bar{x}}_{0}}{\sqrt{\frac{\left(n_{0}-1\right){s_{0}}^{2}+\left(n_{1}-1\right){s_{1}}^{2}}{n_{0}+n_{1}-2}}}\;J \end{array}$$

In this case, ${\bar{x}}_{0}$ represents the mean hyperintensity burden of the control group and ${\bar{x}}_{1}$ that of the patient group within one study, *s*_0_^2^ and *s*_1_^2^ are the corresponding variances and *n*0 and *n*1 represent the number of subjects in each group. For the exact definition of *J* see Hedges (1981).

### Statistical Analyses

All statistical analyses were carried out using RStudio, version 1.0.153. Meta-analytic models were calculated using the R package metafor (Viechtbauer, 2010). Statistical analysis of heterogeneity and inspection of data strongly suggested applying a random-effects model (Cooper, 2009) since the *Q*-statistic revealed a significant heterogeneity between studies (*p* < 0.001) and study characteristics (design, demographics, and methods) were considerably diverging. A multilevel model (Van den Noortgate et al., 2013) was calculated to control for dependencies between outcomes with random effects on the levels outcome nested in sample nested in study taking into consideration that one study may include several samples or several outcome measures within one sample. Thus, it is accounted for that different outcomes from the same sample or study do not represent independent data. The influence of moderating variables on the effect size was investigated in subgroup analyses by including it in the model and testing subgroups against each other via linear hypothesis. Metric variables were investigated by calculating a meta-regression analysis of Hedges' *g*. The significance level for all statistical analyses was set α = 0.05. It is referred to two-sided *p*-values in every case to determine significance.

Outcomes were regarded as outliers if the value deviated more than three standard deviations from the arithmetic mean of Hedges' *g*, as such a deviation is considered highly unlikely under normal distribution (cf. three-sigma rule, Pukelsheim, 1994).

Publication bias was analyzed in three different ways. Firstly, the funnel plot was visually inspected to detect asymmetry. Secondly, as suggested by Sterne and Egger (2005), a meta-regression of the effect size against its variance was conducted, that is, a multilevel random effects meta-regression of Hedges' *g* against its variance was calculated. Thirdly, it was investigated if the binary control variable research interest (i.e., whether hyperintensities were part of the primary research question or not) acted as a moderating factor. This was controlled for by conducting a subgroup analysis.

Interrelation of variables was investigated by computing an interrelation matrix using adequate effect sizes for each interrelation (i.e., Pearson's *r* for two metric variables, Cramer's *V* for two factors, and η^2^ if one variable was metric and the other a factor). Interrelations were regarded as substantial if *r* ≥ 0.05 or if the correlation was significant at α = 0.05 significance level, if *V* ≥ 0.40, and if η^2^ ≥ 0.14 (Cohen, 1977). Each pair of highly confounded variables was included in a multilevel random effects model to test if the effect of one variable is explained by confounding with another variable.

## Results

The demographic characteristics of the included studies (*n* = 68) and samples (*n* = 87) are summarized in Table 1. Patients and controls were of similar age (61.2 and 61.0 years, respectively) and sex (38.5 and 38.0% male, respectively). The characteristics of each sample are listed in Table 2. The data of 3,096 patients and 4,071 controls was included in the present meta-analysis. Table 3 shows the random effects model using Hedges' *g*, which displays the difference in hyperintensity burden between patients and controls across all lesion locations. In accordance with hypothesis 1, the patient group (MDD and BD) exhibited a significantly greater mean severity of hyperintensity burden than the control group (*g* = 0.229, *SE* = 0.031, *p* < 0.001). In this model, three outliers were excluded. This affected the study by Rej et al. (2014) and two outcomes of the study by Tighe et al. (2012) with effect sizes of *g* = −0.918, *g* = 2.293, and *g* = 3.058, respectively. The random effects model including all outcomes (*g* = 0.238, *SE* = 0.042, *p* < 0.001) is shown in Table 4. For all following analyses, outliers were excluded.

**Table 1:** Demographic characteristics of included studies and samples.

	**Mean**	**Min**.	**Max**.	**Mdn**.	**NA%**
Year of publication	2007	1991	2017	2007	0.0
**Patients (*****n*** **=** **3,906)**					
*n* per sample (*SD*)	44.9 (43.3)	10.0	253.0	35.0	0.0
Age (*SD*)	61.2 (15.4)	14.6	75.8	68.8	1.1
Sex, % male (*SD*)	38.5 (15.8)	0.0	100.0	35.9	5.7
**Controls (*****n=*** **4,071)**					
*n* per sample (*SD*)	46.8 (42.8)	11.0	270.0	32.0	0.0
Age (SD)	61.0 (15.0)	16.0	74.9	69.1	1.1
Sex, % male (*SD*)	38.0 (15.4)	0.0	100.0	38.0	4.6

*Outliers were excluded, n, number of subjects; Mdn., median; NA% percentage of missing data on outcome level*.

**Table 2:** Sample Characteristics.

		**Patients**				**Controls**			
**References**	**Dis**.	***n***	**Age**	***SD***	**Onset**	***n***	**Age**	***SD***	**Lesion location**
Agid et al., 2003	MDD	37	55.0	16.3	NS	27	50.0	11.2	OWMH
Ahn et al., 2004	BD	43	36.9	11.5	NS	39	35.1	9.7	DWMH, PVH
Allan, 2014	MDD	32	68.9	5.7	LL	94	69.2	5.3	DWMH, PVH
Alves et al., 2012	MDD	17	65.5	5.5	LL	18	66.4	3.5	OWMH
Birner et al., 2015	BD	100	44.0	14.0	NS	54	41.0	16.0	OWMH
Brown et al., 1992	MDD	28	40.3	10.5	NS	154	34.0	9.5	DWMH, PVH
Chen C. S. et al., 2006	MDD	14	75.1	6.3	LO	11	72.6	8.1	OWMH
Colloby et al., 2011	MDD	38	74.1	6.1	LL	30	74.4	6.4	DWMH, PVH
Davis et al., 2004	BD	22	43.1	11.2	NS	32	37.8	10.8	OWMH
De Asis et al., 2006	BD	40	69.8	6.7	LL	15	66.9	6.5	DWMH, SCGMH
Delaloye et al., 2010	MDD	30	65.0	4.2	EO	30	70.8	6.8	DWMH, PVH, SCGMH
Delaloye et al., 2010	MDD	11	75.8	6.5	LO	30	70.8	6.8	DWMH, PVH, SCGMH
El-Badri et al., 2006	BD	50	30.2	6.2	NS	26	30.2	6.2	DWMH, PVH
Emsell et al., 2017	MDD	55	73.6	7.4	LL	52	72.7	6.5	OWMH
Feng et al., 2014	MDD	57	71.8	5.0	EO	85	72.3	5.9	DWMH, PVH
Feng et al., 2014	MDD	84	73.2	4.8	LO	85	72.3	5.9	DWMH, PVH
Feng et al., 2014	MDD	54	73.1	5.6	NS	85	72.3	5.9	DWMH, PVH
Firbank et al., 2004	MDD	29	75.7	5.9	LL	32	74.9	7.0	OWMH, DWMH, SCGMH
Gildengers et al., 2015	BD	58	64.5	9.8	LL	21	65.6	7.5	OWMH
Greenwald et al., 1998	MDD	35	74.7	6.4	LL	31	72.9	4.7	DWMH, SCGMH
Greenwald et al., 1996	MDD	48	74.6	6.1	LL	39	72.6	6.4	DWMH, PVH, SCGMH
Greenwald et al., 2001	MDD	40	74.6	6.2	LL	21	74.6	5.8	DWMH, SCGMH
Greenwald et al., 2001	MDD	41	74.7	6.2	LL	49	71.9	5.5	DWMH, SCGMH
Gunning-Dixon et al., 2010	MDD	22	69.7	4.7	LL	25	70.7	5.8	Other
Harada et al., 2016	MDD	45	60.2	8.2	LL	61	62.9	7.6	DWMH, PVH, SCGMH
Hickie et al., 2005	MDD	47	52.8	12.6	LL	21	54.7	9.1	DWMH, PVH, SCGMH
Hong et al., 2009	MDD	178	69.9	7.7	LL	85	70.0	5.7	OWMH, GMH
Iosifescu et al., 2005	MDD	50	14.6	10.3	NS	35	39.2	9.8	DWMH, PVH
Iosifescu et al., 2006	MDD	19	39.6	7.6	NS	35	39.2	9.8	DWMH, PVH
Iosifescu et al., 2006	MDD	65	40.7	10.2	NS	35	39.2	9.8	DWMH, PVH
Johnson et al., 2017	MDD	130	69.9	7.0	LL	110	70.2	5.8	OWMH
Keshavan et al., 1996	MDD	19	67.0	5.6	LL	19	64.2	8.3	DWMH, PVH, SCGMH
Kieseppä et al., 2014	BD	13	42.8	11.1	NS	21	41.7	11.3	DWMH
Kieseppä et al., 2014	BD	15	38.4	9.1	NS	21	41.7	11.3	DWMH
Kieseppä et al., 2014	MDD	16	48.4	10.3	NS	21	41.7	11.3	DWMH
Köhler et al., 2011	MDD	20	70.9	6.4	LL	25	72.0	6.1	OWMH
Köhler et al., 2010	MDD	35	74.1	6.5	LL	29	72.8	6.9	OWMH
Kramer-Ginsberg et al., 1999	MDD	41	74.6	6.0	LL	38	72.8	6.4	DWMH, PVH, SCGMH
Kumar et al., 2000	MDD	51	74.3	6.6	LL	30	69.4	6.1	Other
Lee et al., 2003	MDD	38	68.7	7.0	LL	41	71.2	6.3	DWMH, SCGMH
Lenze et al., 1999	MDD	24	52.8	18.4	NS	24	52.7	18.4	DWMH, PVH, SCGMH
Lesser et al., 1996	MDD	60	63.1	7.9	LO	165	64.4	9.6	OWMH
Lesser et al., 1996	MDD	35	57.3	6.1	EO	165	64.4	9.6	OWMH
Lin et al., 2005	MDD	37	72.7	3.6	LO	18	70.2	4.7	DWMH, PVH
Lloyd et al., 2004	MDD	23	72.7	6.7	EO	39	73.1	6.7	DWMH, PVH
Lloyd et al., 2004	MDD	28	75.1	5.8	LO	39	73.1	6.7	DWMH, PVH
Lloyd et al., 2009	BD	48	44.5	8.9	NS	47	45.8	8.6	DWMH, PVH, SCGMH
Madsen et al., 2012	MDD	14	56.0	NA	EO	14	63.0	NA	DWMH, PVH
Madsen et al., 2012	MDD	14	67.0	NA	LO	14	63.0	NA	DWMH, PVH
Mak et al., 2016	MDD	33	73.6	5.2	LL	25	73.6	6.0	DWMH, PVH
McDonald et al., 1991	BD	12	68.3	7.0	LO	12	68.7	7.0	OWMH
McDonald et al., 1999	BD	38	35.9	11.0	LO	30	34.4	8.0	DWMH, PVH, SCGMH
McDonald et al., 1999	BD	32	68.2	9.0	LL	40	67.3	7.0	DWMH, PVH, SCGMH
Miller et al., 1994	MDD	19	69.0	6.0	LL	23	68.0	8.0	DWMH, PVH, SCGMH
Moore et al., 2001	BD	15	42.1	13.9	NS	15	41.9	12.6	DWMH, PVH
Moore et al., 2001	BD	14	47.4	10.1	NS	15	41.9	12.6	DWMH, PVH
O'Brien et al., 1996	MDD	60	71.2	7.9	NS	39	71.4	11.0	DWMH, PVH
Oh and Cheon, 2004	MDD	32	63.1	10.0	LL	25	60.5	6.6	DWMH, PVH
Paranthaman et al., 2010	MDD	25	72.6	4.4	LL	21	72.2	6.5	DWMH, PVH, SCGMH
Patel et al., 2012	MDD	33	68.3	6.6	LL	41	71.7	7.9	OWMH
Payne et al., 2013	MDD	29	68.3	6.5	LL	13	68.3	6.5	OWMH
Pillai et al., 2002	BD	15	15.0	2.4	Ped.	16	16.0	1.8	OWMH
Potter et al., 2007	MDD	83	71.6	6.3	LL	47	74.1	6.4	Other
Rabins et al., 1991	MDD	21	NA	NA	LL	16	NA	NA	OWMH, PVH, SCGMH
Rolstad et al., 2016	BD	75	36.5	12.2	NS	83	38.6	14.5	DWMH
Sassi et al., 2003	BD	24	34.2	9.9	NS	38	36.8	9.7	DWMH, PVH, SCGMH
Sassi et al., 2003	MDD	17	42.8	9.2	NS	38	36.8	9.7	DWMH, PVH, SCGMH
Schwichtenberg et al., 2017	MDD	45	73.7	6.3	LO	42	72.0	6.7	Other
Sexton et al., 2012	MDD	36	71.8	7.7	LL	25	71.8	7.3	DWMH, PVH
Silverstone et al., 2003	BD	13	40.2	NA	NS	19	35.9	NA	DWMH, PVH
Silverstone et al., 2003	MDD	11	34.4	NA	EO	19	35.9	NA	DWMH, PVH
Tamashiro et al., 2008	BD	10	73.6	4.1	LO	24	69.0	7.2	DWMH, PVH, SCGMH
Tamashiro et al., 2008	BD	49	67.8	4.4	EO	24	69.0	7.2	DWMH, PVH, SCGMH
Taylor et al., 2007	MDD	226	70.0	7.4	LL	144	70.3	6.5	OWMH, SCGMH
Taylor et al., 2005	MDD	253	70.5	6.2	LL	146	69.9	7.5	OWMH, SCGMH
Taylor et al., 2014	MDD	47	69.3	6.5	LL	70	69.7	6.2	OWMH
Taylor et al., 2014	MDD	18	70.0	6.1	LL	70	69.7	6.2	OWMH
Taylor et al., 2014	MDD	27	72.1	7.3	LL	70	69.7	6.2	OWMH
Taylor et al., 2013b	MDD	54	68.9	5.6	LL	37	73.8	5.8	OWMH
Taylor et al., 2008	MDD	199	70.0	7.8	LL	113	69.9	5.6	OWMH, SCGMH
Tighe et al., 2012	BD	12	33.8	2.4	NS	31	33.0	2.0	OWMH
Tupler et al., 2002	MDD	69	70.5	8.1	LO	37	65.9	9.4	DWMH, PVH, SCGMH
Tupler et al., 2002	MDD	46	61.1	12.2	EO	37	65.9	9.4	DWMH, PVH, SCGMH
Vasudev et al., 2012	MDD	41	74.0	5.9	LL	32	74.6	6.3	DWMH, PVH
Weber et al., 2010	MDD	38	66.1	6.2	EO	62	71.1	7.3	DWMH, PVH, SCGMH
Wu et al., 2014	MDD	65	73.2	4.4	LO	270	72.3	5.7	OWMH

*Each sample is listed once. Dis., Disorder; Age, age (M); SD, age (SD); Onset, age at illness onset categorically assigned to the following groups; NS, not specified; LL, late-life depression; LO, late-onset depression; EO, early-onset depression; Ped., pediatric depression; OWMH, overall white matter hyperintensities; DWMH, deep white matter hyperintensities; PVH, periventricular hyperintensities; SCGMH, subcortical gray matter hyperintensities*.

**Table 3:** Random effects model excluding 3 outliers.

***g***	***n***	***s***	***o***	***SE***	**95% CI**	***p*-value**	**τ**
0.229	68	87	202	0.031	[0.168, 0.290]	< 0.001	0.225

*n, number of studies; s, number of samples; o, number of outcomes; g, standardized mean difference Hedges' g; SE, standard error; CI, confidence interval at 95% level of significance; τ, tau (estimate of the total standard deviation of the true effect size Hedges' g)*.

**Table 4:** Random effects model including outliers.

***g***	***n***	***s***	***o***	***SE***	**95 % CI**	***p*-value**	**τ**
0.238	69	90	205	0.042	[0.157, 0.320]	< 0.001	0.439

*g, standardized mean difference Hedges' g; SE, standard error; CI, confidence interval at 95% level of significance; τ, tau (estimate of the total standard deviation of the true effect size Hedges' g)*.

Visual inspection of the forest plot revealed a substantial heterogeneity between outcomes, which was also reflected by the estimated total standard deviation of the true effect from the multilevel random effects model as τ = 0.225 (Table 3).

### Subgroup Analyses

Table 5 displays the subgroup analyses. The mean severity of hyperintensity burden was greater in either patient sample compared with the control group when including the subgroups MDD and BD in the model (*p* < 0.001 for both subgroups). The BD subgroup exhibited a significantly greater effect than the MDD subgroup (*p* = 0.012).

**Table 5:** Subgroup analyses.

							***p*****-values of subgroup analyses**			
**Subgroup**	***n***	***s***	***o***	***g***	***SE***	***p*-value**	**1 vs. 2**	**2 vs. 3**	**1 vs. 3**	**τ**
Disorder										0.218
1. MDD	54	66	151	0.189	0.034	<0.001	0.012	–	–	
2. BD	17	21	51	0.374	0.066	<0.001	–	–	–	
Onset group										0.154
1. Late-onset	13	13	33	0.560	0.060	<0.001	<0.001	–	–	
2. Early-onset	9	9	27	−0.031	0.066	0.644	–	0.008	–	
3. Late-life	37	37	98	0.169	0.037	<0.001	–	–	<0.001	
Lesion assessment										0.223
1. Rating scale	42	57	159	0.246	0.038	<0.001	0.053	–	–	
2. Automated	13	15	21	0.085	0.075	0.258	–	0.027	–	
3. Semi-automated	12	13	18	0.327	0.079	<0.001	–	–	0.352	

*n, number of studies; s, number of samples; o, number of outcomes; g, Hedges' g; MDD, major depressive disorder; BD, bipolar disorder; τ, tau (estimate of the total standard deviation of the true effect size) for each model including one moderator, respectively*.

As displayed in Table 5, a late illness onset (late-onset depression) was associated with a greater hyperintensity burden than early-onset depression (*p* < 0.001), which is in line with hypothesis 2. Further, the late-onset depression group exhibited a significantly greater effect size than the late-life depression group (*p* < 0.001). These effects were also evident when investigating the interaction model including disorder (subgroups MDD and BD) and onset (subgroups early-onset, late-onset, and late-life). That is, both in unipolar and bipolar depression, late-onset depression was associated with a greater difference in hyperintensity burden between patients and controls than early-onset or late-life depression (Table 6).

**Table 6:** Subgroup analyses for interaction model including disorder and onset group.

							***p*****-values of subgroup-analyses for subgroups 1-3**			
**Interaction**	***n***	***s***	***o***	***g***	***SE***	***p*-value**	**1 vs. 2**	**2 vs. 3**	**1 vs. 3**	**τ**
										0.143
**MDD**										
1 Late-onset	10	10	21	0.516	0.067	<0.001	<0.001	–	–	
2 Early-onset	8	8	20	−0.081	0.071	0.255	–	0.003	–	
3 Late-life	34	38	89	0.159	0.038	<0.001	–	–	<0.001	
**BD**										
1 Late-onset	3	3	12	0.766	0.131	<0.001	<0.001	–	–	
2 Early-onset	1	1	7	0.211	0.153	0.170	–	0.497	–	
3 Late-life	3	3	9	0.334	0.121	0.006	–	–	0.003	

*n, number of studies; s, number of samples; o, number of outcomes; g, Hedges' g; MDD, major depressive disorder; BD, bipolar disorder; τ, tau (estimate of the total standard deviation of the true effect size) for the interaction model including the moderators disorder and onset*.

Investigating lesion location (DWMH, PVH, and SCGMH), Hedges' *g* turned out significant in each subgroup regarding the whole patient sample (*p* < 0.001 for each subgroup). The interaction model including lesion location and disorder (MDD and BD) revealed that Hedges' *g* was significant in all lesion location subgroups in MDD. In BD, however, the effect size for SCGMH did not reach significance (*p* = 0.071). All other *p*-values for lesion location subgroups in MDD and BD ranged from *p* < 0.001 to *p* = 0.014. In subgroup analysis, no significant differences between DWMH, PVH, and SCGMH in effect size were evident regarding the whole patient group, as well as regarding MDD. In BD, on the contrary, Hedges' *g* was significantly greater for DWMH than for PVH (*p* = 0.033) and SCGMH (*p* = 0.005).

### Control Variables

Three control variables turned out significant. Firstly, subgroup analysis revealed that the type of lesion assessment had a significant influence (Table 5). Semi-automated lesion assessment was associated with a significantly greater effect size than automated lesion assessment (*p* = 0.027). However, no difference in effect size was observed between volumetric outcome measures and severity ratings using semi-quantitative rating scales (*p* = 0.559).

Secondly, meta-regression analysis revealed a significant influence of age (*p* < 0.001) and age at illness onset (*p* = 0.037). Age was defined as the difference in years between the patient and the control sample, thus, a higher age in the patient group compared to the control group was significantly associated with a more pronounced difference in hyperintensity burden. An increase in age at illness onset was also significantly associated with a greater effect size. No other control variable included in the coding scheme turned out significant in meta-regression or subgroup analysis. The *p*-values ranged from *p* = 0.059 (hypertension) to *p* = 0.854 (smoking).

### Interrelations Between Variables

Table 7 displays the measures of interrelation between variables. Substantial interrelations were found between onset and disorder, onset and age, as well as between lesion assessment and lesion location, which means that MDD and BD were not equally distributed across the onset groups, that the age difference between patients and controls differed between the onset groups, and that the lesion locations were not equally distributed across the lesion assessment categories. Each pair of interrelated variables was included in a multilevel random effects model. None of the models did differ from the above-mentioned outcome patterns, which means that interrelation of variables did not confound effect sizes, with one exception: Table 5 displays a significant difference between early-onset and late-life depression (*p* = 0.008). When including age in the model, this difference is no longer significant (*p* = 0.125). Notably, the differences between late-onset and early-onset depression, as well as between late-onset and late-life depression remain significant when controlling for age (*p* < 0.001 in both cases).

**Table 7:** Interrelations between variables.

	**Disorder**	**Lesion assessment**	**Lesion location**	**Primary res**.	**Age^**i**^**
Onset	0.409	0.224	0.198	0.249	0.209
Lesion assessment	0.145	–	0.447	0.196	0.009
Lesion localization	0.122	–	–	0.315	0.009
Primary res.	<0.001	–	–	–	0.018
Age^i^	0.013	–	–	–	–

*Pearson's r, Cramer's V and η^2^ used where adequate, Primary res., primary research question (whether hyperintensities were related to the primary research question), ^i^age difference in years between the patient and the control sample*.

### Publication Bias

**Figure 2:**
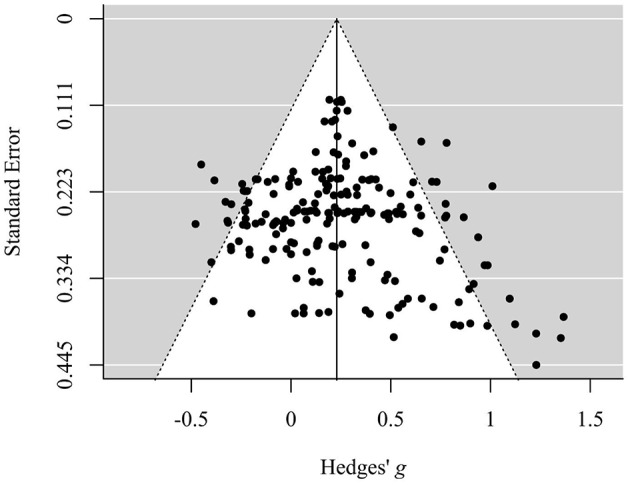
Funnel plot displaying the distribution of standard errors in dependence of corresponding standardized mean differences (Hedges' *g*). Data is shown on outcome level.

Visual inspection of the funnel plot allows for a first evaluation regarding the existence of publication bias (Egger et al., 1997). The absence of publication bias is assumed to reflect the following pattern: The effect sizes of samples with a large sample size (and therefore small *SE*) are distributed near the average, while effect sizes of small samples (and therefore large *SE*) reveal a more widespread distribution, which leads to a symmetric, funnel-shaped distribution of effect sizes around the average. In this case, visual inspection of the funnel plot (Figure 2) suggested a slight asymmetry with missing outcomes in the lower left sections, that is, outcomes with an effect size that is smaller than the average and with a larger *SE* (i.e., smaller sample size) seemed to be underrepresented.

However, the meta-regression analysis of Hedges' *g* against its variance revealed a small and non-significant intercept (*p* = 0.910), which indicates that there is no evidence for funnel plot asymmetry as the regression line runs through zero. More precisely, on the basis of this model a “small study effect” is unlikely. This indicates that the meta-analytic model is not biased by a selective publication of studies in dependence of effect size and *SE* in view of the fact that studies with a small number of participants (and therefore a larger *SE*) are commonly more likely to be publicized if they show a greater effect size in the desired direction.

Another aspect of possible publication bias refers to the study aims. The binary control variable research interest differentiated whether hyperintensity burden was part of the primary research question. Hedges' *g* was significantly greater in studies in which hyperintensity burden was part of the primary research question (*p* = 0.029). This might hint at a publication bias in the sense that studies in which hyperintensity burden is the core object of investigation are more likely to be publicized if the effect size is substantial. This would not affect studies that regard hyperintensity burden as a secondary outcome with respect to study aims. Collectively, the presence of a publication bias cannot be conclusively excluded.

## Discussion

### Summary of Main Findings

Including 68 studies, the meta-analysis represents the most extensive overview of the state of research regarding the severity of hyperintensity burden in MDD (*n* = 54) and BD (*n* = 17) in comparison to healthy controls so far. To a large extent, both hypotheses were confirmed by the statistical analyses: Hyperintensity burden was higher in the entire depression group compared to the control group, which was also evident within each depression group (MDD and BD) and within the categories DWMH, PVH, and SCGMH (hypothesis 1), though the analyses did not confirm an association of SCGMH burden with BD. The association between hyperintensity burden and depression was moderated by age at illness onset in terms of late-onset depression being associated with a more pronounced difference in hyperintensity burden between patients and controls in comparison to early-onset depression in both MDD and BD (hypotheses 2). Notably, it is the first meta-analysis to demonstrate a significant difference between late-life and late-onset depression and, in terms of the outcome measure, this is the first meta-analysis that investigated the association between BD and hyperintensity severity burden instead of frequency. Regarding MDD, this was conducted by three prior meta-analyses (Herrmann et al., 2008; Kempton et al., 2011; Anorne et al., 2012). However, the present meta-analysis provides a more comprehensive overview of the current state of research on hyperintensity severity in MDD as a substantially greater number of studies were included in the analyses. Taken together, the results underscore the association of hyperintensities with unipolar and bipolar depression, which is in line with prior meta-analyses (Beyer et al., 2009; Wang et al., 2014; van Agtmaal et al., 2017).

The results reveal a substantial heterogeneity with τ = 0.225 in relation to *g* = 0.229, which has to be taken into account when interpreting the results. This heterogeneity is reduced to τ = 0.127 by including the moderators disorder, onset, and age, which shows that these variables explain heterogeneity to some degree. However, its magnitude suggests that the association of MDD and BD with hyperintensities varies across conditions and cannot be assumed to predict lesion load on an individual level unrestrictedly.

### Linking Hyperintensities and Depression

#### Hyperintensities Displaying Disruption in White Matter Tracts

Hyperintense lesions are linked to decreased fractional anisotropy (FA) and an increased apparent diffusion coefficient (ADC), which is evident in healthy as well as in depressed subjects (Taylor et al., 2001; Vernooij et al., 2008). For example, impaired integrity in tracts that connect prefrontal regions and subcortical gray matter structures might thus be presented as DWMH on MRI scans. Abnormalities in these tracts are assumed to interrupt brain circuitries involved in emotion processing and are therefore assumed to play a role in MDD and BD symptomatology (Mahon et al., 2010).

#### Hyperintensity-Related Correlates on Molecular Level

Recent approaches explaining the association between lesion load and depression depict a less deterministic perspective than the vascular depression hypothesis. However, these are closely related to the vascular depression hypothesis, since they likewise refer to the association between vascular changes, lesion load, and depression. On the molecular level, three interrelated mechanisms are suggested (Santos et al., 2012). Firstly, an elevated plasma homocysteine level is associated with hyperintense lesions and is assumed to be a risk factor for depression in later life (Almeida et al., 2007; Kim et al., 2008; Tseng et al., 2009). One of the possible mechanisms explaining this association is that elevated homocysteine levels can lead to endothelial dysfunction (Tawakol et al., 1997; Lentz, 2005). Endothelial dysfunction, in turn, is proposed to represent the second mechanism linking hyperintensities and depression. Several markers of endothelial dysfunction are associated with unipolar and bipolar depression (Rybakowski et al., 2006) as well as with white matter hyperintensities (Zupan et al., 2015). There is evidence for a bidirectional relationship between depressive symptoms and endothelial dysfunction (Kim et al., 2010). The third mechanism linking hyperintensities and depression considers inflammatory activity. For example, Interleukin 6 (IL-6) was shown to be associated with late-life depression (Penninx et al., 2003; Bremmer et al., 2008) as well as with DWMH and PVH (Nagai et al., 2011). IL-6 is, like homocysteine, assumed to induce endothelial dysfunction (Wassmann et al., 2004). Therefore, these models presume an interaction between several hyperintensity-related correlates of depression on molecular level and do not exclude a bidirectional relation of depression and lesion load. For a more detailed overview, see Santos et al. (2012).

### Hyperintensity Burden in MDD vs. BD

Several studies indicate that the frequency of subjects with hyperintensities is higher in BD compared to MDD (Dupont et al., 1995; Kieseppä et al., 2014), while other studies did not find such a difference (Sassi et al., 2003). Here it was shown that subjects with BD exhibit a more severe hyperintensity burden than subjects with MDD. Specifically, the significant role of DWMH in BD compared to PVH and SCGMH seems to distinguish hyperintensity burden in BD from that of MDD, as in MDD no difference in lesion load regarding DWMH, PVH, and SCGMH was observed. The more pronounced DWMH burden in BD compared to MDD might be explained by differences in the extent of decreased FA between MDD and BD. The meta-analysis by Nortje et al. (2013) demonstrated a broad white matter involvement in BD patients regarding changes in diffusion properties. One study compared whole-brain FA between MDD subjects, BD subjects, and healthy controls (Versace et al., 2010). In BD, bilateral changes in white matter connectivity (i.e., decreased FA) were found in regions involved in emotion regulation and sensory processing, while in MDD, only unilateral changes were evident. It is suggested that these more global abnormalities in white matter tracts in BD compared to MDD patients might account for the characteristic mood lability in BD (Cardoso de Almeida and Phillips, 2013).

Another possible explanation is that the more pronounced hyperintensity burden in BD, compared to MDD, does not cause bipolar symptomatology, but rather reflects the presence of hyperintensity-related correlates that are more prevalent in BD than in MDD, for example, obesity, metabolic syndrome, and cardiovascular risk factors such as hypertension (Fiedorowicz et al., 2008). This assumption seems plausible, as these risk factors are associated with hyperintensities (Portet et al., 2012; Yin et al., 2014; Habes et al., 2016). According to this view, hyperintense lesions are indicators for the presence of comorbidities which frequently occur in the course of BD (Gunde et al., 2011).

Furthermore, the more pronounced lesion load regarding DWMH compared to PVH in BD might be explained on molecular level. Permoda-Osip et al. (2013) demonstrated that hyperhomocysteinemia frequently occurs in BD subjects during an acute affective episode. In addition, elevated homocysteine levels are associated with common comorbidities of BD such as hypertension or metabolic syndrome (Sutton-Tyrrell et al., 1997; Hajer et al., 2007). Regarding lesion location, there are several studies showing that elevated homocysteine levels are rather linked to DWMH than to PVH (Hogervorst et al., 2002; Sachdev, 2004; Sachdev et al., 2004). Therefore, a high prevalence of hyperhomocysteinemia in BD patients might to some degree account for the difference between DWMH and PVH burden which was found in the present meta-analysis.

### Theoretical Implications: Confining the Vascular Depression Hypothesis

The present meta-analysis underscores the relevance of age at illness onset with regard to lesion load. Consistent with prior findings (Herrmann et al., 2008), hyperintensity burden is more severe in late-onset depression in comparison to early-onset depression, that is, in the present study, the difference in hyperintensity burden between patients and controls is more pronounced in late-onset depression compared to early-onset depression. The findings demonstrate that this pattern is evident in both unipolar and bipolar depression. Therefore, the results can be interpreted as underlining the significance of cerebrovascular correlates when it comes to depression in the elderly.

The association of vascular lesions with depressive symptomatology in older age was first described in 1905 (Gaupp, 1905) and referred mainly to arteriosclerotic phenomena. By introducing the vascular depression hypothesis, Alexopoulos et al. (1997) presented a more elaborate concept which involves cerebrovascular changes. According to this theory, disruption in brain circuits that subserve mood regulation due to cerebrovascular lesions is the core mechanism in the etiology of vascular depression in later life. In the case of BD, some authors refer to this concept using the term *vascular mania* (Steffens and Krishnan, 1998). As mentioned before, the vascular depression hypothesis is not restricted to late-onset depressive syndromes, arguing that depression in earlier life increases the risk for vascular diseases which, in turn, can lead to vascular depression in later life (Taylor et al., 2013a). In view of the present findings, however, it seems reasonable to link the concept of vascular depression specifically to late-onset rather than late-life depression. Although, late-life depression with an early illness onset might in some cases likewise be of a vascular type, this phenomenon can be assumed to be significantly less common—at least when referring to hyperintensities as the hallmark of vascular depression—as demonstrated by the findings of the present meta-analysis: Firstly, it is shown that hyperintensity burden is significantly more severe in late-onset depression than in late-life depression, which was observed in the entire patient sample, as well as in MDD and BD. This difference might be due to the circumstance that late-life depression can comprise both early- and late-onset depression which are in many cases not distinguished between when investigating late-life depression (van Agtmaal et al., 2017), while late-onset depression is restricted to late-life depression with a late illness onset (Aizenstein et al., 2016). It can therefore not be excluded that the association of late-life depression with hyperintensities might rather be explained, i.e., mediated by late-onset depression. Secondly, when controlling for age, there was no longer a significant difference between late-life and early-onset depression, while the differences between late-onset and late-life depression as well as between late-onset and early-onset depression remained significant. Vascular depression among early-onset patients is unlikely, as there was no significant difference regarding hyperintensity burden in the early-onset subgroup compared to the control group. In addition, age at illness onset turned out to be a significant moderator. Taken together, these findings provide evidence that the vascular depression subtype is more likely when the first episode occurs in older age.

In conclusion, it might be more feasible to focus on late-onset depression when it comes to defining vascular depression. Confining the concept on the basis of the above delineated evidence might allow for more precise theoretical elaboration and research on vascular depression. With respect to specifying diagnostic criteria for the vascular subtype of depression, a stricter definition (i.e., a late illness onset) helps to increase its internal and external validity and might, on that account, subserve its diagnostic implementation in clinical context. Why identifying a vascular subtype of depression might be of practical relevance concerning individualized treatment planning, is discussed below. Taken together, vascular depression might in some cases have an early illness onset, but this circumstance does not justify the rather vague focus on late-life depression, which is currently more common in research—as also displayed by the number of studies included in the different onset subgroups of the present meta-analysis. In this view, the present results strongly suggest confining the concept of vascular depression to late-onset depression in terms of its feasibility in research as well as in clinical context.

Although the present meta-analysis is in line with the vascular depression hypothesis regarding the association of hyperintensities with depression in the elderly, it should not be mistaken as fostering this theory taking into account the cross-sectional nature of the meta-analysis. No conclusions on causal directions can be drawn. Therefore, strictly speaking, the findings should not be construed as proving evidence for the vascular depression hypothesis as such, viz. that cerebrovascular disease may entail depressive disorders in old age (predispose, precipitate, or perpetuate, cf. (Alexopoulos et al., 1997), which would at least to some degree imply causality.

### Neuropathological Correlates of Late-Onset Depression

A broader understanding of neuropathological changes in late-onset depression is essential to determine its etiopathogenetic factors. In addition to hyperintensity burden, there is a variety of abnormalities associated with late-onset depression, as demonstrated by a substantial number of studies. This underlines the clinical validity of distinguishing late-onset depression from depression in earlier life. With regard to vascular burden, Smith et al. (2009) found that a later age at illness onset in depressed subjects were associated with higher levels of intima-media thickness. Intima-media thickness, in turn, was shown to correlate with white matter hyperintensities in late-onset depression (Chen C. S. et al., 2006). Further, Liao et al. (2017) found that late-onset depression is characterized by greater abnormalities in cerebral blood flow compared to early-onset depression. Regarding neurostructural changes, Xekardaki et al. (2012) report in their review that volume reductions in several brain regions occur in late-onset depression in comparison to both healthy control subjects and subjects with early-onset depression. Specifically, studies on structural brain changes found that late-onset depression was associated with right frontal lobe atrophy (Almeida et al., 2003), gray matter reductions in parahippocampal area, parietal inferior area, cingulum and putamen (Andreescu et al., 2008), and decrease of volume in the right rostral hippocampus, the right amygdala, and the medial orbito-frontal cortex (Egger et al., 2008). Comparing late-onset depression with late-life-early-onset depression, Disabato et al. (2014) found that late-onset depression was associated with a smaller left anterior cingulate thickness. Further neuroanatomical changes in the limbic system are described by Choi et al. (2017) who demonstrated total hippocampal and hippocampal subfield volume reductions in subjects with late-onset depression in comparison to healthy controls. Interestingly, hippocampal subfield volumes were partly correlated with white matter hyperintensity volume. Concerning neurofunctional changes, studies using functional MRI (fMRI) or positron-emission tomography (PET) reveal several abnormalities in patients with late-onset depression. Fujimoto et al. (2008) describe widespread changes in the distribution of metabolism in subjects with treatment-resistant late-onset depression compared to age-matched healthy controls. Alterations were not only evident in limbic circuits, but also in a wider range of thalamo-cortical circuits. By measuring regional homogeneity in resting-state fMRI signals in depressed, treatment-naive subjects, Chen et al. (2012) found differences in regional brain activity between late-onset and early-onset depression in the right precuneus and bilateral superior frontal gyrus. Recently, Liu et al. (2018) identified abnormal functional connectivity in anterior and posterior sub-networks of the default mode network as a potential risk factor for late-onset depression. Taken together, neuropathological correlates of late-onset depression seem to comprehend multiple cerebrovascular, neurostructural and neurofunctional changes compared to both healthy control subjects and subjects with early-onset depression.

However, there are several studies that do not confirm that late-onset depression is characterized by distinct neuropathological phenomena (Santos et al., 2010; Jellinger, 2011; Dols et al., 2017). As a possible explanation for the non-significant association between late-onset depression and microvascular disease shown in several post-mortem studies, Xekardaki et al. (2012) point out the relevance of psychosocial determinants in late-onset depression. The contradictory findings could also be interpreted as demonstrating that late-onset depression is not necessarily of a vascular subtype. This would be in line with the substantial heterogeneity found in the present study. In view of the multiple neuropathological phenomena that are found in late-onset depression it remains to be investigated how these are related to vascular depression. The diverse abnormalities on cerebrovascular, neurostructural, and neurofunctional level indicate that late-onset depression represents a heterogeneous disease pattern, within which vascular depression may be one possible subtype.

### Limitations

There are some limiting factors that should be taken into account when interpreting the results. Above all, the cross-sectional nature of the analyses must be considered when referring to implications of the findings on theoretical and practical level. Therefore, on the basis of the present meta-analysis, no conclusions on causality regarding the association between depression and hyperintensity burden can be drawn. All explanations and implications of findings that are depicted in this article display only a few of many possible other mechanisms and consequences.

Several control variables that can be assumed to be associated with hyperintensities were included. Except for age, none of these turned out significant. The non-significant findings, however, may be the consequence of inappropriate or insufficient data provided by the primary studies. This affects the variables hypertension, diabetes mellitus, depression severity, smoking, and medication. Therefore, the analyses on control variables do not represent the whole dataset, and confounding by such variables that, in most cases, were not reported in primary studies can—despite of the non-significant findings—not be excluded.

In addition, the interaction model for disorder and onset includes only a limited number of studies in the BD onset-subgroups. This might limit the validity of the depicted outcome pattern.

A bias due to selective publication is unlikely, but cannot be excluded. Controlling for the role of hyperintensities regarding the research question(s) of the primary studies revealed that studies in which the focus lies on investigating hyperintensities show a greater effect size. In view of this, a publication bias might exist in the sense that studies with smaller effect sizes were less likely to be publicized if hyperintensity burden was the main outcome measure.

A further limitation regards the database literature search. Even though the present meta-analysis comprises a substantial number of studies and, therefore, is likely to represent a comprehensive overview of the present state of research, it cannot be excluded that a more elaborate literature search including further databases (e.g., Scopus) might have yielded even more potentially relevant studies. However, due to limited resources, the literature search was restricted to the above mentioned databases.

### Future Research

The present meta-analysis focuses on severity ratings and volumetric measurements as indicators of the extent of hyperintensity burden in patients and controls, while prior meta-analyses predominantly compared the frequency of subjects with hyperintensities between both groups. As hyperintensities are a common phenomenon of old age (de Leeuw et al., 2012), rather the extent of hyperintensity burden than the number of subjects with hyperintensities might represent a meaningful parameter when investigating depression in later life. Future research might directly compare severity ratings with frequency ratings to examine potential differences between these two outcome measures.

Regarding age at illness onset, the studies included in the present meta-analysis use different cut-offs to differentiate between early-onset and late-onset depression. It seems unlikely that a single cut-off age can be determined. However, future research regarding late-onset depression or vascular depression, respectively, could engage in identifying an age at onset range which is most likely to indicate the presence of a vascular subtype of depression.

The role of hyperintensity assessment requires further research. While there was no significant difference in effect size between volumetric outcomes and severity measurements using semi-quantitative rating scales, fully-automated vs. semi-automated lesion assessment did make a difference within volumetric measurements. Hyperintensity burden was significantly higher when lesion volume was determined using semi-automated procedures compared to automated (Table 5). At first glance, this might hint at some kind of researcher bias. Yet, the binary control variable blinding did not turn out significant (*p* = 0.885). Thus, the reason for the effect of automated vs. semi-automated hyperintensity assessment remains unclear. Aside from bias, it is possible that this difference reflects a distinctive characteristic of depression-related hyperintensities. The nature of hyperintense lesions that are associated with depression might be specific in such a way that they cannot be adequately detected using fully automated assessment processes, e.g., because they are too small or the sensitivity of automated algorithms is too low. In this respect, it should be considered to which extent, i.e., limits, segmentation criteria applied by a radiologist or expert rater can be incorporated into fully automated segmentation algorithms. This further questions the feasibility of fully automated lesion assessment procedures in general. These and other possible explanations need to be scrutinized in future research.

### Clinical Implications

The pronounced difference in hyperintensity burden comparing late-onset to early-onset depression in MDD and BD suggests a clinical differentiation according to age at illness onset. Nevertheless, this does not mean that late-onset depression is accompanied by cerebrovascular pathology in every case. A more reasonable explanation would be that among late-onset depression the vascular subtype is more common, that is, late-onset depression is more frequently but not necessarily associated with a substantial lesion load.

There is evidence that vascular MDD is associated with increased functional impairment relative to non-vascular MDD (González et al., 2012), which underscores the clinical relevance of distinguishing between vascular and non-vascular depression. This differentiation in clinical context gains importance when it comes to treatment planning. For example, it might be worth considering a co-treatment of cerebrovascular pathology in addition to treating the mood disorder alone. In this respect, a thorough examination of vascular risk factors that possibly underlie hyperintense lesions can facilitate individualized treatment options for vascular depression. First of all, this affects medication. In late-onset depressive syndromes, not only psychoactive drugs, but also those that might prevent or reduce hyperintensity progression should be examined for possible benefits regarding depression treatment, e.g., cilostazol (Takahashi and Mikuni, 2012) or other substances that improve cerebral blood flow. Moreover, hyperintensity-related correlates on molecular level could be taken into account when considering broader medical treatment options, for example, with respect to inflammatory processes (Müller et al., 2006). In clinical context, however, side-effects of such additional medical treatment must be considered.

In addition, the benefits of different classes of antidepressant drugs should be taken into account. As mentioned above, vascular depression is characterized, among others, by a reduced response rate to antidepressant medication and by cognitive decline. This raises the question if antidepressant drugs that show effects on neurostructural and neurofunctional level can enhance response rates to medical treatment of vascular depression. For example, Pompili et al. (2013) report in their review that agomelatine, an atypical antidepressant drug targeting melatonergic receptors, is not only effective with regard to its antidepressant action and side-effect profile, but also promotes neurogenesis in the hippocampus and the prefrontal cortex as well as enhancing neuroplasticity mechanisms. Whether these neurofunctional and neurostructural effects can improve treatment response in vascular depression would thus be an interesting question regarding medical treatment options.

Aside from medical conditions, lifestyle aspects can lead to or increase cerebrovascular lesion burden, such as smoking or obesity (Zhu et al., 2014; Habes et al., 2016). Therefore, treatment of vascular depression might profit from an approach tackling behavioral patterns that are associated with progressing cerebrovascular pathology.

In the present meta-analysis, age (i.e., the difference in years between the patient and the control sample) turned out to be a significant moderator. This underlines the general association of age with hyperintensities (de Leeuw et al., 2012), which might be explained by vascular changes associated with aging (Marín, 1995; Kovacic et al., 2011) and the higher prevalence of vascular-related diseases in the elderly (Kearney et al., 2005; Shaw et al., 2010). Nevertheless, even though hyperintensities are a common condition in later life, they might represent a premorbid phenotype of depression. For example, the meta-analysis by Kloppenborg et al. (2014) demonstrated that WMH progression was associated with decline in several cognitive domains which, in turn, represents a characteristic symptomatology in geriatric depression, especially in late-onset depression (Lesser et al., 1996; Rapp et al., 2005). The association of hyperintensities with age and depression as well as with abnormalities in cognitive functioning (as shown by Kloppenborg et al., 2014) underlines their clinical relevance in geriatric healthcare. For example, depression screenings in later life that give attention to the symptom classes of late-onset depression might be useful to detect premorbid vascular depression.

### Final Conclusions

To conclude, there is an association between hyperintensities and depression which seems to be strongly dependent on age at illness onset. On the basis of the present findings it seems reasonable to assume that hyperintensity-related depression is most likely to be characterized by a late illness onset. This association should be taken into account when investigating etiologic models for MDD and BD in later life. Specifically, it suggests to confine the concept of vascular depression to late-onset depression. This confinement seems feasible in a research as well as in a clinical context. Within a research context it might enhance internal and external validity when it comes to elaborating the concept of vascular depression. In a clinical context, the confinement gains importance regarding diagnostic criteria and the detection of vascular depression. A depression diagnosis that differentiates vascular from non-vascular depression might allow for a more individualized treatment plan. This, finally, might reduce the rate of treatment-resistant depression in later life. On the one hand, this would affect the choice of antidepressant medication as well as considering other potentially adjuvant drugs. Aside from medication, behavioral patterns associated with hyperintensities likewise merit consideration in the context of vascular depression treatment. The moderating role of age regarding hyperintensity burden suggests that the risk for vascular depression is increased in the elderly, at least when assuming that hyperintensities represent a preceding or interacting risk factor for vascular depression. Thus, treating vascular diseases and reducing vascular risk factors that are associated with hyperintensities could help to prevent depressive syndromes in the elderly.

Overall, future research should consider different perspectives regarding possible mechanisms underlying the relationship between hyperintensities and depression in order to achieve a broader understanding of illness predisposition, triggering, perpetuation, and consequences. This, essentially, might allow for more individualized and potent treatment options regarding depressive disorders.

## Author Contributions

KS and IW conducted the literature search. KS coded the studies. KS and JS performed the data analysis. KS, JS, RS, HH, and IW wrote the article.

### Conflict of Interest Statement

The authors declare that the research was conducted in the absence of any commercial or financial relationships that could be construed as a potential conflict of interest.
